# Redetermination of (*E*)-*N*,*N*′-bis­(4-bromo­phen­yl)formamidine

**DOI:** 10.1107/S1600536811013419

**Published:** 2011-04-16

**Authors:** L.-J. Han

**Affiliations:** a Department of Chemistry, Tongji University, Shanghai 200092, People’s Republic of China

## Abstract

In comprison with the previous structural study [Anulewicz *et al.* (1991 ▶). *Pol. J. Chem.* **65**, 465–471], for which only the coordinates of all non-H atoms and of some H atoms were reported, the current redetermination of the title compound, C_13_H_10_Br_2_N_2_, additionally reports anisotropic displacement parameters for all non-H atoms and the coordinates of all H atoms, accompanied by higher accuracy of the geometric parameters. Two independent half-mol­ecules are present in the asymmetric unit, which are completed by a twofold rotation axis as symmetry element. In the crystal, inter­molecular N—H⋯N hydrogen bonds link the mol­ecules into dimers. Linear chains parallel to [102] are formed by inter­molecular Br⋯Br inter­actions of 3.4328 (7) Å between two Br atoms of adjacent mol­ecules. The dihedral angles between the benzene rings are 50.05 (15) and 75.61 (11)° in the two independent molecules. Owing to the twofold symmetry of the mol­ecules, H atoms attached to the N atoms are only half-occupied, leading to them being disordered over two positions of equal occupancy.

## Related literature

For the previous structure determination, see: Anulewicz *et al.* (1991 ▶). For Br⋯Br inter­actions, see: Fujiwara *et al.* (2006 ▶); Reddy *et al.* (1996 ▶). For N—H⋯N hydrogen bonds, see: Del Bene & Elguero (2006 ▶); Grotjahn *et al.* (2000 ▶); Thar & Kirchner (2006 ▶).
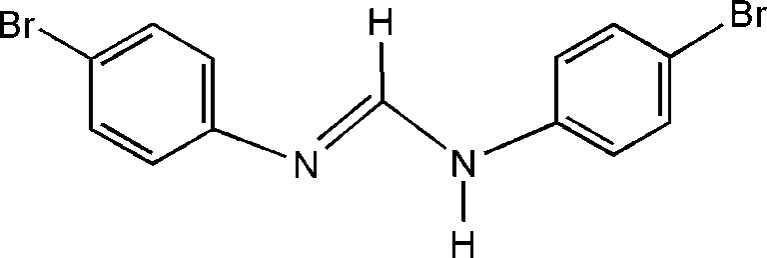


## Experimental

### 

#### Crystal data

C_13_H_10_Br_2_N_2_
*M* *_r_* = 354.05
Monoclinic, 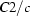

*a* = 11.563 (2) Å
*b* = 23.447 (5) Å
*c* = 9.881 (2) Å
β = 95.43 (3)°
*V* = 2666.9 (9) Å^3^
*Z* = 8
Mo *K*α radiation
μ = 6.06 mm^−1^
*T* = 293 K
0.15 × 0.07 × 0.06 mm

#### Data collection

Bruker SMART CCD diffractometer
Absorption correction: multi-scan (*SADABS*; Sheldrick, 2004 ▶) *T*_min_ = 0.403, *T*_max_ = 0.695
5954 measured reflections
2611 independent reflections
1715 reflections with *I* > 2σ(*I*)
*R*_int_ = 0.061

#### Refinement

*R*[*F* ^2^ > 2σ(*F* ^2^)] = 0.048
*wR*(*F* ^2^) = 0.114
*S* = 1.00
2611 reflections
155 parameters
H-atom parameters constrained
Δρ_max_ = 0.48 e Å^−3^
Δρ_min_ = −0.91 e Å^−3^

### 

Data collection: *APEX2* (Bruker, 2004 ▶); cell refinement: *SAINT-Plus* (Bruker, 2001 ▶); data reduction: *SAINT-Plus*; program(s) used to solve structure: *SHELXS97* (Sheldrick, 2008 ▶); program(s) used to refine structure: *SHELXL97* (Sheldrick, 2008 ▶); molecular graphics: *XP* (Sheldrick, 2008 ▶) and *DIAMOND* (Brandenburg, 1999 ▶); software used to prepare material for publication: *SHELXL97*.

## Supplementary Material

Crystal structure: contains datablocks global, I. DOI: 10.1107/S1600536811013419/wm2476sup1.cif

Structure factors: contains datablocks I. DOI: 10.1107/S1600536811013419/wm2476Isup2.hkl

Additional supplementary materials:  crystallographic information; 3D view; checkCIF report

## Figures and Tables

**Table 1:** Hydrogen-bond geometry (Å, °)

*D*—H⋯*A*	*D*—H	H⋯*A*	*D*⋯*A*	*D*—H⋯*A*
N1—H2*A*⋯N2^i^	0.85	2.12	2.964 (4)	180
N2—H3*A*⋯N1^ii^	0.88	2.12	2.964 (4)	161

Symmetry codes: (i) 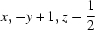; (ii) 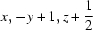.

**Fig. 1.**
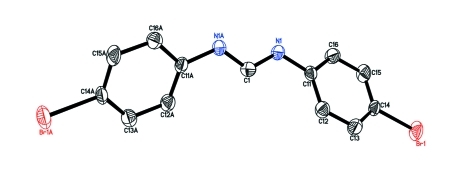
Molecular structure of one of the two molecules of (I) drawn with displacement ellipsoids at the 30% probability level. [Symmetry code A) -x+1, y. -z+1/2.]

**Fig. 2.**
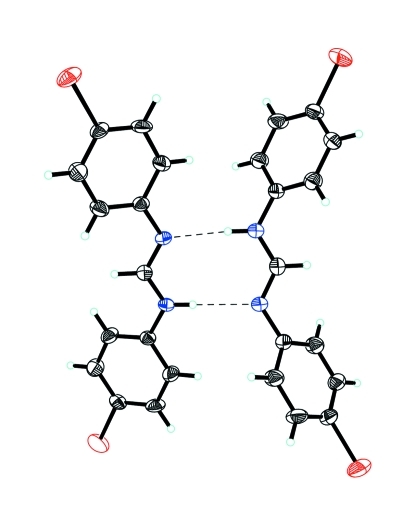
A dimer formed by intermolecular N–H···N hydrogen bonds.

**Fig. 3.**
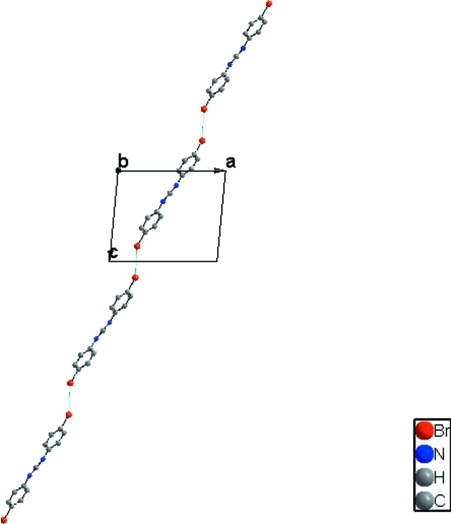
Part of an one-dimensional linear chain of the title compound, viewed along [010]. Br···Br interactions are drawn with blue dashed lines.

## supplementary crystallographic information

### Comment

With the determination of reliable intermolecular distances, Br···Br interactions (Fujiwara *et al.*, 2006; Reddy *et al.*, 1996) and N–H···N hydrogen bonding (Del Bene & Elguero, 2006; Grotjahn *et al.* 2000; Thar & Kirchner, 2006) became important criteria in the description of supramolecular chemistry and in applied crystal engineering. The title compound C_13_H_10_Br_2_N_2_, (I), has been determined previously by Anulewicz *et al.* (1991). However, in that study only coordinates of all non-H atoms and of some H atoms were given. The present re-determination additionally reports anisotropic displacement parameters for all non-H atoms and the coordinates of all H atoms, accompanied by higher accuracy of all geometric parameters.

In (I) two independent half-molecules are present in the asymmetric unit which are completed by a twofold rotation axis as symmetry element that runs to the central C—H groups (C1—H1 and C2—H2, respectively). One molecule is displayed in Fig. 1. The dihedral angles between the two benzene rings in the individual molecules are 50.05 (15) ° for the first and and 75.61 (11) ° for the second molecule.

In the crystal, intermolecular N—H···N hydrogen bonds link the individual molecules into dimers (Fig. 2). Linear chains parallel to [102] are formed by intermolecular Br···Br interactions of 3.4328 (7) Å between two bromine atoms of adjacent molecules (Fig. 3). This interaction is significantly less than the van der Waals contact of 3.90 Å (Reddy *et al.*, 1996; Fujiwara *et al.*,  2006), hence making this interaction important for consolidation of the crystal packing.

### Experimental

The title compound was synthesized by the following reaction. 17.202 g (0.1 mol) of 4-bromobenzenamine and 8.33 ml (0.05 mol) of triethyl orthoformate were combined in a round-bottom flask equipped with a distillation tube and heated at 160 until the distillation of ethanol creased. The retained solid was washed with ether, and dried under a dynamic vacuum to yield 16.10 g of white solid, (91%). 0.04 g of the white solid was dissolved in THF (3 ml) and the solution was layered with hexane. Colourless needle-shaped crystals formed after several days. ^1^HNMR(CDCl_3_, p.p.m.): 8.08(s, 1H, –NCHN–), 7.43(d, 2H, aromatic), 7.40(d, 2H, aromatic), 6.93(d, 2H, aromatic), 6.91(d, 2H, aromatic). Anal. Calcd. C_13_H_10_Br_2_N_2_: C, 44.10; H, 2.85; N, 7.91; Found: C, 43.83; H, 2.69; N, 8.02.

### Refinement

H atoms attached to C atoms were positioned geometrically with C—H = 0.93 (CH), and constrained to ride on their parent atoms, with *U*_iso_(H) = 1.2*U*_eq_(C). H atoms attached to N atoms were found from difference Fourier maps and were fixed. They were refined with *U*_iso_(H) = 1.2*U*_eq_(N). Owing to the 2 symmetry of the molecules, the H atoms attached to the N atoms are only half-occupied, leading to being disordered over two positions of equal occupancy.

### Crystal data

**Table:**

C_13_H_10_Br_2_N_2_	*F*(000) = 1376
*M**_r_* = 354.05	*D*_x_ = 1.764 Mg m^−^^3^
Monoclinic, *C*2/*c*	Mo *K*α radiation, λ = 0.71073 Å
Hall symbol: -C 2yc	Cell parameters from 4303 reflections
*a* = 11.563 (2) Å	θ = 2.5–26.7°
*b* = 23.447 (5) Å	µ = 6.06 mm^−^^1^
*c* = 9.881 (2) Å	*T* = 293 K
β = 95.43 (3)°	Needle, colourless
*V* = 2666.9 (9) Å^3^	0.15 × 0.07 × 0.06 mm
*Z* = 8	

### Data collection

**Table:**

Bruker SMART CCD diffractometer	2611 independent reflections
Radiation source: fine-focus sealed tube	1715 reflections with *I* > 2σ(*I*)
graphite	*R*_int_ = 0.061
ω scans	θ_max_ = 26.0°, θ_min_ = 1.7°
Absorption correction: multi-scan (*SADABS*; Sheldrick, 2004)	*h* = −12→14
*T*_min_ = 0.403, *T*_max_ = 0.695	*k* = −26→28
5954 measured reflections	*l* = −12→11

### Refinement

**Table:**

Refinement on *F*^2^	Primary atom site location: structure-invariant direct methods
Least-squares matrix: full	Secondary atom site location: difference Fourier map
*R*[*F*^2^ > 2σ(*F*^2^)] = 0.048	Hydrogen site location: inferred from neighbouring sites
*wR*(*F*^2^) = 0.114	H-atom parameters constrained
*S* = 1.00	*w* = 1/[σ^2^(*F*_o_^2^) + (0.0533*P*)^2^] where *P* = (*F*_o_^2^ + 2*F*_c_^2^)/3
2611 reflections	(Δ/σ)_max_ = 0.001
155 parameters	Δρ_max_ = 0.48 e Å^−^^3^
0 restraints	Δρ_min_ = −0.91 e Å^−^^3^

### Special details

**Table:**

Geometry. All e.s.d.'s (except the e.s.d. in the dihedral angle between two l.s. planes) are estimated using the full covariance matrix. The cell e.s.d.'s are taken into account individually in the estimation of e.s.d.'s in distances, angles and torsion angles; correlations between e.s.d.'s in cell parameters are only used when they are defined by crystal symmetry. An approximate (isotropic) treatment of cell e.s.d.'s is used for estimating e.s.d.'s involving l.s. planes.
Refinement. Refinement of *F*^2^ against ALL reflections. The weighted *R*-factor *wR* and goodness of fit *S* are based on *F*^2^, conventional *R*-factors *R* are based on *F*, with *F* set to zero for negative *F*^2^. The threshold expression of *F*^2^ > σ(*F*^2^) is used only for calculating *R*-factors(gt) *etc*. and is not relevant to the choice of reflections for refinement. *R*-factors based on *F*^2^ are statistically about twice as large as those based on *F*, and *R*- factors based on ALL data will be even larger.

### Fractional atomic coordinates and isotropic or equivalent isotropic displacement parameters (Å^2^)

**Table:**

	*x*	*y*	*z*	*U*_iso_*/*U*_eq_	Occ. (<1)
Br1	0.24399 (5)	0.73912 (2)	0.82770 (5)	0.0708 (2)	
N1	0.4449 (3)	0.63281 (13)	0.3396 (3)	0.0429 (8)	
H2A	0.4352	0.5975	0.3271	0.052*	0.50
C1	0.5000	0.6600 (2)	0.2500	0.0428 (13)	
H1	0.5000	0.6997	0.2500	0.051*	
C11	0.3994 (4)	0.65996 (16)	0.4505 (4)	0.0404 (9)	
C12	0.4347 (4)	0.71277 (17)	0.4995 (4)	0.0525 (11)	
H12A	0.4903	0.7329	0.4570	0.063*	
C13	0.3885 (4)	0.73617 (18)	0.6108 (5)	0.0569 (11)	
H13A	0.4131	0.7719	0.6428	0.068*	
C14	0.3065 (4)	0.70681 (17)	0.6738 (4)	0.0459 (10)	
C15	0.2716 (4)	0.65412 (19)	0.6293 (4)	0.0560 (11)	
H15A	0.2171	0.6340	0.6737	0.067*	
C16	0.3177 (4)	0.63066 (17)	0.5178 (4)	0.0546 (11)	
H16A	0.2935	0.5947	0.4873	0.066*	
Br2	0.02928 (6)	0.61567 (3)	1.05388 (8)	0.1036 (3)	
N2	0.4121 (3)	0.49146 (13)	0.7963 (3)	0.0507 (8)	
H3A	0.4085	0.4541	0.7936	0.061*	0.50
C2	0.5000	0.5180 (2)	0.7500	0.0520 (15)	
H2B	0.5000	0.5577	0.7500	0.062*	
C21	0.3242 (4)	0.52137 (16)	0.8556 (4)	0.0455 (10)	
C22	0.2110 (4)	0.50352 (19)	0.8320 (5)	0.0588 (12)	
H32A	0.1933	0.4723	0.7758	0.071*	
C23	0.1229 (4)	0.53143 (19)	0.8908 (5)	0.0635 (12)	
H33A	0.0466	0.5189	0.8746	0.076*	
C24	0.1488 (4)	0.57764 (18)	0.9729 (5)	0.0561 (11)	
C25	0.2603 (4)	0.59524 (19)	0.9991 (4)	0.0584 (12)	
H35A	0.2774	0.6264	1.0557	0.070*	
C26	0.3483 (4)	0.56719 (17)	0.9422 (4)	0.0561 (11)	
H36A	0.4247	0.5791	0.9621	0.067*	

### Atomic displacement parameters (Å^2^)

**Table:**

	*U*^11^	*U*^22^	*U*^33^	*U*^12^	*U*^13^	*U*^23^
Br1	0.0872 (4)	0.0873 (4)	0.0409 (3)	0.0246 (3)	0.0215 (2)	−0.0074 (2)
N1	0.046 (2)	0.0446 (17)	0.0413 (18)	0.0019 (15)	0.0199 (15)	−0.0018 (14)
C1	0.040 (3)	0.042 (3)	0.047 (3)	0.000	0.006 (3)	0.000
C11	0.044 (2)	0.046 (2)	0.033 (2)	0.0046 (18)	0.0111 (17)	0.0051 (17)
C12	0.059 (3)	0.056 (2)	0.045 (2)	−0.014 (2)	0.017 (2)	−0.001 (2)
C13	0.070 (3)	0.056 (2)	0.046 (3)	−0.009 (2)	0.012 (2)	−0.007 (2)
C14	0.056 (3)	0.055 (2)	0.028 (2)	0.010 (2)	0.0129 (18)	−0.0038 (18)
C15	0.053 (3)	0.073 (3)	0.047 (2)	−0.005 (2)	0.029 (2)	0.000 (2)
C16	0.064 (3)	0.051 (2)	0.053 (3)	−0.009 (2)	0.025 (2)	−0.003 (2)
Br2	0.0764 (5)	0.0940 (5)	0.1485 (7)	0.0149 (3)	0.0538 (4)	−0.0264 (4)
N2	0.046 (2)	0.0459 (18)	0.063 (2)	0.0002 (16)	0.0217 (17)	−0.0012 (17)
C2	0.055 (4)	0.043 (3)	0.059 (4)	0.000	0.011 (3)	0.000
C21	0.047 (3)	0.044 (2)	0.047 (2)	0.0014 (18)	0.0127 (19)	0.0011 (18)
C22	0.049 (3)	0.061 (3)	0.067 (3)	−0.010 (2)	0.014 (2)	−0.019 (2)
C23	0.035 (3)	0.078 (3)	0.079 (3)	−0.006 (2)	0.014 (2)	−0.016 (3)
C24	0.052 (3)	0.057 (3)	0.062 (3)	0.011 (2)	0.019 (2)	0.000 (2)
C25	0.057 (3)	0.053 (3)	0.067 (3)	0.000 (2)	0.017 (2)	−0.014 (2)
C26	0.047 (3)	0.059 (3)	0.061 (3)	−0.007 (2)	0.005 (2)	−0.010 (2)

### Geometric parameters (Å, °)

**Table:**

Br1—C14	1.901 (4)	Br2—C24	1.886 (4)
N1—C1	1.305 (4)	N2—C2	1.311 (4)
N1—C11	1.412 (5)	N2—C21	1.407 (5)
N1—H2A	0.8422	N2—H3A	0.8763
C1—N1^i^	1.305 (4)	C2—N2^ii^	1.311 (4)
C1—H1	0.9300	C2—H2B	0.9300
C11—C12	1.377 (5)	C21—C22	1.373 (6)
C11—C16	1.388 (6)	C21—C26	1.385 (5)
C12—C13	1.381 (6)	C22—C23	1.383 (6)
C12—H12A	0.9300	C22—H32A	0.9300
C13—C14	1.369 (6)	C23—C24	1.369 (6)
C13—H13A	0.9300	C23—H33A	0.9300
C14—C15	1.360 (6)	C24—C25	1.355 (6)
C15—C16	1.383 (6)	C25—C26	1.375 (6)
C15—H15A	0.9300	C25—H35A	0.9300
C16—H16A	0.9300	C26—H36A	0.9300
			
C1—N1—C11	123.2 (3)	C2—N2—C21	121.5 (3)
C1—N1—H2A	116.6	C2—N2—H3A	120.0
C11—N1—H2A	120.1	C21—N2—H3A	118.5
N1^i^—C1—N1	121.4 (5)	N2^ii^—C2—N2	123.3 (5)
N1^i^—C1—H1	119.3	N2^ii^—C2—H2B	118.4
N1—C1—H1	119.3	N2—C2—H2B	118.4
C12—C11—C16	118.0 (4)	C22—C21—C26	118.3 (4)
C12—C11—N1	123.9 (4)	C22—C21—N2	119.4 (3)
C16—C11—N1	118.0 (3)	C26—C21—N2	122.2 (4)
C11—C12—C13	120.9 (4)	C21—C22—C23	120.8 (4)
C11—C12—H12A	119.6	C21—C22—H32A	119.6
C13—C12—H12A	119.6	C23—C22—H32A	119.6
C14—C13—C12	120.0 (4)	C24—C23—C22	119.6 (4)
C14—C13—H13A	120.0	C24—C23—H33A	120.2
C12—C13—H13A	120.0	C22—C23—H33A	120.2
C15—C14—C13	120.5 (4)	C25—C24—C23	120.3 (4)
C15—C14—Br1	119.8 (3)	C25—C24—Br2	119.8 (3)
C13—C14—Br1	119.7 (3)	C23—C24—Br2	119.8 (3)
C14—C15—C16	119.6 (4)	C24—C25—C26	120.2 (4)
C14—C15—H15A	120.2	C24—C25—H35A	119.9
C16—C15—H15A	120.2	C26—C25—H35A	119.9
C15—C16—C11	121.1 (4)	C25—C26—C21	120.7 (4)
C15—C16—H16A	119.5	C25—C26—H36A	119.7
C11—C16—H16A	119.5	C21—C26—H36A	119.7

Symmetry codes: (i) −*x*+1, *y*, −*z*+1/2; (ii) −*x*+1, *y*, −*z*+3/2.

### Hydrogen-bond geometry (Å, °)

**Table:**

*D*—H···*A*	*D*—H	H···*A*	*D*···*A*	*D*—H···*A*
N1—H2A···N2^iii^	0.85	2.12	2.964 (4)	180
N2—H3A···N1^iv^	0.88	2.12	2.964 (4)	161

Symmetry codes: (iii) *x*, −*y*+1, *z*−1/2; (iv) *x*, −*y*+1, *z*+1/2.
